# Shakedown Analysis and Experimental Study of Thermal Barrier Coatings

**DOI:** 10.3390/ma15186446

**Published:** 2022-09-16

**Authors:** Jian Sun, Yunhui Chen, Wanzhong Li, Hualong Chen, Hui Liu, Xiaole Cheng

**Affiliations:** 1 School of Mechanical and Electrical Engineering, Xi’an Polytechnic University, Xi’an 710048, China; 2 Xi’an Key Laboratory of Modern Intelligent Textile Equipment, Xi’an 710048, China; 3 School of Mechanical Engineering, Xi’an Shiyou University, Xi’an 710065, China

**Keywords:** thermal barrier coatings, shakedown analysis, stability, thermal shock test

## Abstract

Interfacial stress–strain fields become complicated in thermal barrier coatings (TBCs) under cyclic thermal loading, which affects the stability and spalling failure of TBCs directly. The convex and concave interfacial structures of TBCs were approximated as a multilayer cylinder model, and an analytical method of TBCs for shakedown analysis was established. A series of 8-YSZ TBC specimens were prepared by the plasma spraying process, followed by isothermal and thermal shock tests. The results showed that the stability limit is significantly greater than the elastic limit, the limit for the convex model was higher than that in the concave model, the first failure occurs in the concave area, and the main failure mode of a thermal barrier coating is the appearance of cracks at the interface layer during a thermal shock test. For the coating samples prepared in this study, the stability limits were between 950 °C and 1050 °C, and the validity of the stability limit analysis model of a multilayer structure was verified.

## 1. Introduction

Thermal barrier coatings (TBCs) are widely used in turbines for propulsion and power generation. The ultimate objective of TBC applications is an improvement in fuel efficiency by increasing the operating temperature. This protective coating is also used to prolong the life of turbine components and to improve the reliability of engine components [1,2,3]. There are four primary constituents in a thermal barrier coating system [4]. They comprise (i) the thermal barrier coating (Top coat, TC) itself; (ii) the super-alloy component, treated here as the substrate (Sub); (iii) an aluminum-containing bond coat (BC) located between the Sub and the TC; and (iv) a thermally grown oxide (TGO), predominantly α-alumina, that forms between the TC and the BC. Because of the multilayer features and undulated interfacial microstructures, a complex stress and strain state appears around the interfacial area under thermal loadings, which induces instability in the system and eventually affects the system’s durability [5,6,7,8].

When a structure is subject to cyclic loads, three structural responses may occur: (i) purely elastic deformation, when the magnitude of the load applied is lower than the elastic limit of the structure; (ii) shakedown status, when the load is larger than the elastic limit of the structure but less than the critical limit-shakedown limit; and (iii) non-shakedown status, when the load applied is higher than the shakedown limit and non-restricted plastic flow occurs. If the non-shakedown condition (iii) occurs, the structure may undergo failure as either an incremental collapse (ratcheting) or alternating plasticity (low cycle fatigue). Generally, there are two ways to calculate the shakedown limit: incremental elastic–plastic analysis and shakedown analysis. The incremental elastic–plastic method is based on a calculation of the full history of the stress–strain curve, which involves a large computation cost. The method with the shakedown analysis is a direct approach to calculating the limit load and the stability of the structure. Classical shakedown theorems were initially proposed just for elastic and perfectly plastic materials. Due to the diversity and complex plastic behavior of engineering materials, these shakedown theorems should be extended before application. The stability analysis for a structure under thermal loading is affected by the following factors, a change in the stress field caused by thermal strain, a change in the yield strength and elastic parameters with temperature, and the creep and relaxation under elevated temperatures [9,10]. Therefore, for a TBC stability analysis under cyclic thermal load, considering the limitations of classical stability theory on materials and the complexity of the stress and strain field under cyclic thermal load, the classical stability theorems need to be developed. Additionally, the influence of temperature on the material parameters and yield strength should be considered.

For the relatively simple structures, the calculation methods and extended formulations of stability analysis are given in the literature [11]. The linear programming method was adopted by most researchers to solve the stability limit based on the essence of the stability theorem. Considering the temperature-dependent yield strength, the stability analysis for symmetrical cylindrical and spherical shell structures under an external force and temperature was calculated using the linear programming method in the literature [12]. Chen [13] analyzed the upper and lower limits of a few examples with a temperature-dependent yield strength using the linear matching method. For cylinder structures, Liu [14] performed a stability analysis under the combination of internal pressure and thermal loading, considering the temperature-dependent yield strength. Du [15] proposed a kinematic shakedown theorem derived from the concept of an assumed fictitious yield surface and with the temperature implicitly contained in the theorem. Then, Du [16] presented a new kinematic shakedown theorem without the concept of an assumed yield surface and with the temperature explicitly contained in the new formulation; the relationship between yield strength and temperature was also simplified as being linear. The influence of temperature on yield strength was obviously at the elevated temperature stage but had little effect at low temperatures, which indicates that approximating this relationship as being linear in the whole temperature range may lead to a significant error. For the stability of TBCs, some researchers [6,17,18,19,20,21,22,23] have proposed the concept of “displacement instability” and implemented experimental and simulation analyses on TBCs under cyclic thermal loading; the results showed that oxide growth is the main factor that induces displacement instability. Moreover, the main unstable mechanisms are the ratcheting effect and a volume decrease in the BC layer. Other researchers have also made explored TBC displacement instability. For example, Xu [24] utilized the elastic–plastic finite element method to analyze the stability of TBCs with different irregular interfacial morphologies, and the influence of the yield stress of the BC layer on coating stability was analyzed. Huang [25] performed a finite element analysis of interface displacement instability for the Fecralloy substrate with human-made grooves embedded into its surface, based upon FEA software, and the simulation result showed great agreement with the experimental result. TBC stability is not only related to the interface stability but also closely connected to the whole structure’s performance.

In this study, an analytical method for performing a shakedown analysis of TBCs is proposed. To verify its effectiveness and the rationality of the results, the convex and concave microstructures near the TBC interface are approximately treated as multi-cylinder models, and 8YSZ thermal barrier coatings are prepared by the plasma spraying process. The isothermal thermal shock test is carried out. The comparative test results show that the shakedown limit can easily be solved using the shakedown limit analysis method based on the multi-layer cylinder model and can easily be used for engineering applications. The stability limit of the thermal barrier coating is higher than the elastic design value, and the stability limit of the convex region of the interface is higher than that of the concave region. The structure first fails in the concave region. The larger the base curvature and coating thickness of the cylinder model, the higher the stability limit of the structure. The analysis results are consistent with the existing test results.

## 2. Shakedown Analysis of Cylinder Model

### 2.1. Cylinder Model Derived from the Interfacial Region of TBCs

**Figure 1:**
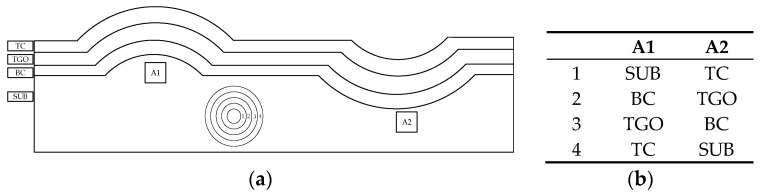
Interfacial geometry of TBCs and schematic of cylinder: (**a**) interfacial geometry of TBCs; (**b**) schematic of cylinder model.

The morphology of complex concave and convex shapes around the TBC interface can be approximately treated as a semicircle, as shown in Figure 1a. Extracting those local characteristics, the cylinder geometry model can be set up. The concave and convex models were built for areas A1 and A2, respectively. The material of each layer is illustrated as shown in Figure 1b. Generally, the thicknesses of TC, BC, and TGO range from 120 to 200 μm, from 30 to 200 μm, and from 2 to 10 μm, respectively [6].

### 2.2. Shakedown Analysis of Multilayer Cylinder Model

For an elasto-perfectly plastic solid body, the volume V, traction ${\hat{p}}_{i}$, and displacement ${\hat{u}}_{i}$ are given on boundaries $S_{P}$ and $S_{u}$. The body force is represented by $Q_{i}$. The basic governing equations and boundary conditions are as follows:

(1) $$\begin{array}{c} \sigma_{ij,j}+Q_{i}=0\;(\mathrm{in}\;V)\\ \varepsilon_{ij}=\left(u_{i,j}+u_{j,i}\right)/2\;(\mathrm{in}\;V)\\ \sigma_{ij}n_{j}={\hat{p}}_{i}\;(\mathrm{on}\;S_{P})\\ u_{i}={\hat{u}}_{i}\;(\mathrm{on}\;S_{u}) \end{array}$$

Considering the geometry and working conditions for TBCs, such as no traction and displacement constraints on the outside surface, the body force, displacement, and strain continuity conditions on the interface can be neglected. Assume that the volume of each layer is $V_{k}$, that the surface of each layer is ${\left(S_{u}\right)}_{k}$, and that the displacement and strain are ${\left({\hat{u}}_{i}\right)}_{k}$ and ${\left({\hat{\varepsilon }}_{i}\right)}_{k}$. Additionally, *k* = 1, 2, 3, 4, which stands for different layers in TBCs, as shown in Figure 1b. Then, the basic governing equations and boundary conditions for TBCs are as follows:

(2) $$\begin{array}{c} {\left(\sigma_{ij,j}\right)}_{k}=0\;(\mathrm{in}\;V_{k})\\ {\left(\varepsilon_{ij}\right)}_{k}=\left({\left(u_{i,j}\right)}_{k}+{\left(u_{j,i}\right)}_{k}\right)/2\;(\mathrm{in}\;V_{k})\\ {\left(\varepsilon_{ij}\right)}_{k}={\left({\hat{\varepsilon }}_{ij}\right)}_{k}\;(\mathrm{on}\;{\left(S_{u}\right)}_{k})\\ {\left(u_{i}\right)}_{k}={\left({\hat{u}}_{i}\right)}_{k}\;(\mathrm{on}\;{\left(S_{u}\right)}_{k}) \end{array}$$

Taking one layer in the multilayer cylinder model as an analysis object, elastic strain $\varepsilon_{ij}^{E}$ and thermal strain $\varepsilon_{ij}^{T}$ are given by

(3) $$\varepsilon_{ij}^{E}=A_{ijkl}\sigma_{kl},\varepsilon_{ij}^{T}=a_{ij}T$$

where $A_{ijkl}$ is a symmetric and positive definite elastic tensor, $a_{ij}$ is a tensor that stands for thermal expansion coefficient, and *T* stands for temperature.

Ignoring the viscosity factor during deformation, the total strain $\varepsilon_{ij}$ is the sum of three components in the following form:

(4) $$\varepsilon_{ij}=\varepsilon_{ij}^{E}+\varepsilon_{ij}^{P}+\varepsilon_{ij}^{T}$$

where $\varepsilon_{ij}^{P}$ is the plastic strain. The yield surface equation is given by

(5) $$F=f\left(\sigma_{ij}\right)-K\left(T\right)=0$$

where $f\left(\sigma_{ij}\right)$ is the stress field and K(T) is the yield stress.

The flow rules are given by [15]

(6) $$\{\begin{array}{l} {\dot{\varepsilon }}_{ij}^{P}=\dot{\lambda }\partial F/\partial \sigma_{ij}\\ \dot{\lambda }\geq 0,if\;F=0\;and\;\frac{\partial f}{\partial \sigma_{ij}}{\dot{\sigma }}_{ij}-\frac{dK}{dT}\dot{T}=0\\ \dot{\lambda }=0,if\;F<0\;or\;F=0\;and\;\frac{\partial f}{\partial \sigma_{ij}}{\dot{\sigma }}_{ij}-\frac{dK}{dT}\dot{T}<0 \end{array}$$

where $\dot{\lambda }$ is the plastic multiplier ratio. Then,

(7) $$\dot{\lambda }\geq 0,\dot{\lambda }F=0$$

The plastic power is defined as

(8) $$D=\sigma_{ij}{\dot{\varepsilon }}_{ij}^{P}=D\left({\dot{\varepsilon }}_{ij}^{P},T\right)$$

Define

(9) $$\{\begin{array}{l} K\left(T\right)=K_{0}h\left(T\right)\\ K_{0}=K\left(0\right) \end{array}$$

where h(T) is a function related to temperature and h(0) = 1.

From Equations (6)–(9), we have

(10) $$D=D_{0}\left({\dot{\varepsilon }}_{ij}^{P}\right)h\left(T\right)$$

where $D_{0}$ is the specific dissipation rate when *T* = 0.

The total stress $\sigma_{ij}^{}$ can be divided into two parts:

(11) $$\sigma_{ij}=\sigma_{ij}^{E}+\rho_{ij}$$

where $\sigma_{ij}^{E}$ is the thermal elastic stress when the structure is treated as an ideal elastic solid under the same external loading and temperature. $\rho_{ij}$ is the residual stress, which is self-equalization stress and only determined by the instantaneous plastic strain field in the structure.

(12) $$\rho_{ij}=L\left(\varepsilon_{ij}^{P}\right)$$

where L is a given linear integral operator.

For simplicity, only consider the quasi-static process here. Assuming that loads and temperature fields are linearized forms of load-temperature coefficients $a_{s}$ (s = 1, 2, …, n_s_), then

(13) $$\sigma_{ij}^{E}\left(r,t\right)=\sum_{s=1}^{n_{s}}a_{s}\left(t\right)\sigma_{ij}^{ES}\left(r\right)$$

where $\sigma_{ij}^{ES}\left(x\right)$ is the thermal elastic stress field when $a_{s}=1$, $a_{j}=0\left(j\neq s\right)$, r is spatial coordinate, and *t* is the time variable. Thus, total strain $\varepsilon_{ij}$ can also be written as

(14) $$\varepsilon_{ij}=A_{ijkl}\sigma_{kl}^{E}+\varepsilon_{ij}^{T}+\varepsilon_{ij}^{P}+A_{ijkl}\rho_{kl}$$

where

(15) $$A_{ijkl}\sigma_{kl}^{E}+\varepsilon_{ij}^{T}=\left(u_{i,j}^{E}+u_{j,i}^{E}\right)/2$$

(16) $$\varepsilon_{ij}^{P}+A_{ijkl}\rho_{kl}=\left(u_{i,j}^{R}+u_{ij,i}^{R}\right)/2$$

(17) $$u_{i}=u_{i}^{E}+u_{i}^{R}$$

where $u_{i}^{E}$ is the thermal elastic displacement field and $u_{i}^{R}$ is the residual displacement.

$u_{i}^{E}$ can be expressed as

(18) $$u_{i}^{E}\left(r,t\right)=\sum_{s=1}^{n}a_{s}\left(t\right)u_{i}^{ES}\left(r\right)$$

where $u_{i}^{ES}\left(r\right)$ is the thermal elastic displacement field when $a_{s}=1$, $a_{j}=0\left(j\neq s\right)$.

Employing this description, the change region of the coefficient $a_{s}$ decides the change limit of external loads and temperature.

(19) $$p_{i}\left(r,t\right)=\sum_{s=1}^{n}a_{s}\left(t\right)p_{i}^{s}\left(r\right),r\in S_{p}$$

(20) $$Q_{i}\left(r,t\right)=\sum_{s=1}^{n}a_{s}\left(t\right)Q_{i}^{s}\left(r\right),r\in V$$

(21) $$T\left(r,t\right)=\sum_{s=1}^{n}a_{s}\left(t\right)T^{s}\left(r\right),r\in V$$

Assuming that ${\dot{\varepsilon }}_{ij}^{*P}$ is a kinematically admissible plastic strain rate in a cycle. Then, the plastic strain increment is given by

(22) $$\Delta {\dot{\varepsilon }}_{ij}^{*P}=\int_{t_{0}}^{t_{0}+\tau }{\dot{\varepsilon }}_{ij}^{*P}dt=\frac{1}{2}\left(u_{i,j}^{*}+u_{j,i}^{*}\right)$$

where $u_{i}^{*}=0$(on $S_{u}$).

The kinematic shakedown theorem can be described as follows: if there exists a load-temperature route $a_{s}\left(t\right)\in G$ and a plastic strain rate in the period cycle ${\dot{\varepsilon }}_{ij}^{*P}$, such that Equation (22) is satisfied and [16]

(23) $$\begin{array}{l} \int_{t_{0}}^{t_{0}+\tau_{s}}\left\{\sum_{s=1}^{n}a_{s}\left(t\right)\left[\int_{V}Q_{i}^{s}{\dot{u}}_{i}^{*}dV+\int_{S_{P}}{\hat{p}}_{i}^{s}{\dot{u}}_{i}^{*}dS\right]+\int_{V}a_{ij}T{\dot{\rho }}_{ij}^{*}dV\right\}dt\\ >\int_{t_{0}}^{t_{0}+\tau }\int_{V}D\left({\dot{\varepsilon }}_{ij}^{*P},T\right)dVdt \end{array}$$

Then, shakedown will not occur. Additionally, G is the variation range of $a_{s}$ in n-dimensional space. $\rho_{ij}^{*}$ denotes the residual stress field associated with the plastic strain field ${\dot{\varepsilon }}_{ij}^{*P}$.

Equation (23) is further simplified. Additionally, Equation (13) is rewritten:

(24) $$\sigma_{ij}^{E}=\sigma_{ij}^{EE}+\rho_{ij}^{T}=\sum_{s=1}^{n_{s}}a_{s}\left(t\right)\left[\sigma_{ij}^{EEs}\left(x\right)+\rho_{ij}^{Ts}\left(x\right)\right]$$

where $\sigma_{ij}^{EEs}$ is the stress, and it is only determined by external loads ${\hat{p}}_{i}$ and $Q_{i}$. The elastic stress field $\sigma_{ij}^{EE}$ is determined if $a_{s}=1$, $a_{j}=0\left(j\neq s\right)$. $\rho_{ij}^{Ts}$ equals the residual stress field caused by temperature T when $a_{s}=1$, $a_{j}=0\left(j\neq s\right)$.

With the principle of virtual power, the third term in the left-hand side of Equation (23) can be rewritten as

(25) $$\int_{V}a_{ij}T{\dot{\rho }}_{ij}^{*}dV=\int_{V}\varepsilon_{ij}^{T}{\dot{\rho }}_{ij}^{*}dV=-\int_{V}\rho_{ij}^{T}A_{ijkl}{\dot{\rho }}_{kl}^{*}dV=\int_{V}\rho_{ij}^{T}{\dot{\varepsilon }}_{ij}^{*P}dV$$

In Equation (23), the preceding two terms in the left-hand side can be transformed. Taking into account Equations (8), (24) and (25),

(26) $$\int_{t_{0}}^{t_{0}+\tau }\int_{V}\sigma_{ij}^{E}\left(r,t\right){\dot{\varepsilon }}_{ij}^{*P}\left(r,t\right)dVdt>\int_{t_{0}}^{t_{0}+\tau }\int_{V}D\left({\dot{\varepsilon }}_{ij}^{*P},t\right)dVdt$$

According to Equation (26), the shakedown criteria for four-layer TBCs can be obtained, namely, four inequations similar to Equation (26), corresponding to TC, TGO, BC, and Sub.

Considering that h(T) is the linear function of *T* for simplicity, we let

(27) h(T)=A−BT

where A and B are material constants; thus, we have

(28) $$D\left({\dot{\varepsilon }}_{ij}^{*P},T\right)=D_{0}\left({\dot{\varepsilon }}_{ij}^{*P}\right)h\left(T\right)=AD_{0}\left({\dot{\varepsilon }}_{ij}^{*P}\right)-BTD_{0}\left({\dot{\varepsilon }}_{ij}^{*P}\right)$$

From the shakedown theorem above, the necessary condition for the shakedown state is immediately obtained. If shakedown occurs, then

(29) $$\eta \int_{t_{0}}^{t_{0}+\tau }\int_{V}\left[\sigma_{ij}^{E}\left(r,t\right){\dot{\varepsilon }}_{ij}^{*P}\left(r,t\right)+BTD_{0}\left({\dot{\varepsilon }}_{ij}^{*P}\right)\right]dVdt\leq \int_{t_{0}}^{t_{0}+\tau }\int_{V}AD_{0}\left({\dot{\varepsilon }}_{ij}^{*P}\right)dVdt$$

where η is the safety factor and η≥1.

Next, the time integration is solved by the incremental failure criterion.

According to Equation (22),

(30) $$\Delta {\dot{\varepsilon }}_{ij}^{*P}\left(r\right)=\varepsilon_{ij}^{*P}\left(r,t_{0}+\tau \right)-\varepsilon_{ij}^{*P}\left(r\right)$$

According to the process and concept of incremental failure, there exists a scalar function λ(r,t) such that

(31) $$\varepsilon_{ij}^{*P}\left(r,t\right)=\lambda \left(r,t\right)\Delta \varepsilon_{ij}^{*P}\left(r\right)$$

where

(32) $$\dot{\lambda }\left(r,t\right)\geq 0,\;\lambda \left(r,t_{0}\right)=0,\lambda \left(r,t_{0}+\tau \right)=1$$

Noticing that the specific dissipation rate $D_{0}\left({\dot{\varepsilon }}_{ij}^{*P}\right)$ is the homogenous function of plastic strain rate ${\dot{\varepsilon }}_{ij}^{*P}$, from Equations (31) and (32), we obtain

(33) $$\int_{t_{0}}^{t_{0}+\tau }D_{0}\left({\dot{\varepsilon }}_{ij}^{*P}\right)dt=\int_{t_{0}}^{t_{0}+\tau }D_{0}\left(\dot{\lambda }\Delta \varepsilon_{ij}^{*P}\right)dt=D_{0}\left(\Delta \varepsilon_{ij}^{*P}\right)$$

From Equations (13), (21) and (23), taking η=1, Equation (29) can be written as

(34) $$\int_{t_{0}}^{t_{0}+\tau }\int_{V}\sum_{s=1}^{n}a_{s}\left(t\right)\left[\sigma_{ij}^{Es}\left(r\right){\dot{\varepsilon }}_{ij}^{*P}\left(r,t\right)+BT^{s}\left(r\right)D_{0}\left({\dot{\varepsilon }}_{ij}^{*P}\right)\right]dVdt\leq \int_{V}AD_{0}\left(\Delta \varepsilon_{ij}^{*P}\right)dV$$

Taking into account Equations (31) and (32), and $D_{0}\left(\dot{\lambda }\Delta \varepsilon_{ij}^{*P}\right)=\dot{\lambda }D_{0}\left(\Delta \varepsilon_{ij}^{*P}\right)$, the left-hand side of Equation (34) can be written as

(35) $$\int_{t_{0}}^{t_{0}+\tau }\int_{V}\sum_{s=1}^{n}a_{s}\left(t\right)\left[\sigma_{ij}^{Es}\left(r\right)\dot{\lambda }\left(r,t\right)\Delta \varepsilon_{ij}^{*P}\left(r\right)+BT^{s}\left(r\right)\dot{\lambda }\left(r,t\right)D_{0}\left(\Delta \varepsilon_{ij}^{*P}\left(r\right)\right)\right]dVdt$$

For the given incremental failure mechanism $\Delta \varepsilon_{ij}^{*P}\left(x\right)$, the function λ(r,t) is chosen by the following method. In region *G* of $a_{s}\left(t\right)$, only if

(36) $$\sum_{s=1}^{n}a_{s}\left(t\right)\left[\sigma_{ij}^{Es}\left(r\right)\Delta \varepsilon_{ij}^{*P}\left(r\right)+BT^{s}\left(r\right)\dot{\lambda }\left(r,t\right)D_{0}\left(\Delta \varepsilon_{ij}^{*P}\left(r\right)\right)\right]$$

achieves admissible maximum can we have $\dot{\lambda }\left(x,t\right)\neq 0$. Now, the left-hand side of Equation (34) can achieve its maximum. Letting

(37) $$f\left(r\right)=\underset{a_{s}\in G}{\max}\left\{\sum_{s=1}^{n}a_{s}\left(t\right)\left[\sigma_{ij}^{Es}\left(r\right)\Delta \varepsilon_{ij}^{*P}\left(r\right)+BT^{s}\left(r\right)D_{0}\left(\Delta \varepsilon_{ij}^{*P}\left(r\right)\right)\right]\right\}$$

be equal to $D_{0}\left(\Delta \varepsilon_{ij}^{*P}\left(r\right)\right)$, the incremental failure criterion is obtained. Lastly, the incremental failure criterion can be expressed in the following form:

(38) $$\int_{V}f\left(r\right)dV=\int_{V}AD_{0}\left(\Delta \varepsilon_{ij}^{*P}\right)dV$$

The variation range G of $a_{s}$ is defined as a group of inequalities:

(39) $$a_{s}^{-}\leq a_{s}^{}\leq a_{s}^{+}$$

Then, Equation (38) can be written as

(40) $$\int_{V}\sum_{s=1}^{n}\beta_{s}\left(r\right)y_{s}\left(r\right)dV=\int_{V}AD_{0}\left(\Delta \varepsilon_{ij}^{*P}\right)dV$$

where $\beta_{s}\left(r\right)$ and $y_{s}\left(r\right)$ are given by

(41) $$\{\begin{array}{l} \beta_{s}\left(r\right)=\left\{ \begin{array}{l} a_{s}^{+},y_{s}\left(r\right)>0\\ a_{s}^{-},y_{s}\left(r\right)<0 \end{array}\right.\\ y_{s}\left(r\right)=\sigma_{ij}^{Es}\left(r\right)\Delta \varepsilon_{ij}^{*P}\left(r\right)+BT^{s}\left(r\right)D_{0}\left(\Delta \varepsilon_{ij}^{*P}\left(r\right)\right) \end{array}$$

Then, the shakedown limit can be obtained from Equations (40) and (41).

## 3. Shakedown Analysis for Typical TBCs

### 3.1. Material Model and Loading

The bilinear plastic properties for BC are listed in Table 1, which appears to be temperature-dependent [26,27]. The yield strength of TGO remains at 10 GPa between 0 and 800 °C but decreases linearly to 2 GPa at 1000 °C [28]. Temperature shows less effects on yield strength for BC and TGO under low temperatures, but the yield strength is significantly reduced above 800 °C. Therefore, it is not accurate to simplify the relationship between yield strength and temperature as being linear in the whole load range. The TC and Sub layers are treated as elastic, and the specified parameters can be found in the literature [29]. Considering the temperature-dependent property of TBCs, the various physical parameters are converted to the function of external load *T* by curve fitting, so that the analytic mathematical model derived above can be used for the stability limit solution. The radiuses of the interface from inside to outside are set to 5 mm, 5.13 mm, 5.135 mm, and 5.165 mm.

The thermal load is applied to the surface of TC, and the cool gas flows inside the Sub to reduce the system temperature. The thermal stress calculating condition is set at 1000 °C on TC and at 25 °C on the Sub inner side. 

Therefore, for a load-temperature coefficient $a_{s}$, the thermal load ranges from 25 °C to 1000 °C and ΔT lies within 0–975 °C, which corresponds to $a_{s}$ being 0–1.

Assuming that the material of TGO and BC conforms to the Tresca yield criterion, the yield stress decreases as the temperature increases.

(42) $$\max\left\{\left|\sigma_{r}|,|\sigma_{\varphi }|,|\sigma_{\varphi }-\sigma_{r}\right|\right\}=2K\left(T\right)$$

where

(43) $$K\left(T\right)=K_{0}\left(A-BT\right),\;\left(2K_{0}=\sigma_{s}\right)$$

### 3.2. Bilinear Model for Yield Strength and Temperature

The radial velocity for small deformation can be described as $\dot{u}\left(r\right)=\frac{\dot{c}}{r}$.

When the incremental collapse is studied, we have

(44) $$\{\begin{array}{l} \Delta \varepsilon_{r}=\int_{t_{0}}^{t_{0}+\tau }{\dot{\varepsilon }}_{r}dt=\int_{t_{0}}^{t_{0}+\tau }\frac{\partial \dot{u}}{\partial r}dt=-\frac{\Delta c}{r^{2}}\\ \Delta \varepsilon_{\varphi }=\int_{t_{0}}^{t_{0}+\tau }{\dot{\varepsilon }}_{\varphi }dt=\int_{t_{0}}^{t_{0}+\tau }\frac{\dot{u}}{r}dt=\frac{\Delta c}{r^{2}} \end{array}$$

where Δc>0. $D_{0}\left(\Delta {\dot{\varepsilon }}_{ij}^{*P}\right)$ refers to the above plastic deformation state and can be expressed as

(45) $$D_{0}=\sigma_{r}\left(-\frac{\Delta c}{r^{2}}\right)+\sigma_{\varphi }\left(\frac{\Delta c}{r^{2}}\right)=\frac{\Delta c}{r^{2}}\left(\sigma_{\varphi }-\sigma_{r}\right)=2K_{0}\frac{\Delta c}{r^{2}}$$

It is necessary to evaluate $\sigma_{ij}^{Es}\left(x\right)\Delta \varepsilon_{ij}^{*P}\left(x\right)$ in $y_{s}\left(r\right)$ to calculate the left-hand side in Equation (40).

(46) $$\sigma_{ij}^{Es}\left(r\right)\Delta \varepsilon_{ij}^{*P}\left(r\right)=\sigma_{r}^{E}\left(r\right)\Delta \varepsilon_{r}\left(r\right)+\sigma_{\varphi }^{E}\left(r\right)\Delta \varepsilon_{\varphi }\left(r\right)$$

where $\sigma_{r}^{E}\left(r\right)$ is the radial stress and $\sigma_{\varphi }^{E}\left(r\right)$ is the circumferential stress, which can be obtained from the literature [30,31].

Since $y_{s}\left(r\right)=\sigma_{ij}^{Es}\left(r\right)\Delta \varepsilon_{ij}^{*P}\left(r\right)+BT^{s}\left(r\right)D_{0}\left(\Delta \varepsilon_{ij}^{*P}\left(r\right)\right)>0$ is always satisfied, $\beta_{s}\left(r\right)=1$.

Transforming Equation (40), we obtain

(47) $$A_{D}=\int_{V}D_{0}\left(\Delta {\dot{\varepsilon }}_{ij}^{*P}\right)dV-\int_{V}\sum_{s=1}^{n}\beta_{s}\left(r\right)y_{s}\left(r\right)dV=\int_{a_{i}}^{a_{i+1}}D_{0}\left(\Delta {\dot{\varepsilon }}_{ij}^{*P}\right)rdr-\int_{a_{i}}^{a_{i+1}}\sum_{s=1}^{n}\beta_{s}\left(r\right)y_{s}\left(r\right)rdr$$

The expression for each variable in Equation (47) can be obtained, and substituting thermal load $T_{w}$ into Equation (47), a function with only one variable ($T_{w}$) can be derived. Due to *A_D_* being characterized as a decreasing function, when the temperature is set initially at 25 °C, the function’s value is greater than zero. Therefore, $A_{D}$ decreases with increasing $T_{w}$, and when $A_{D}$ is equal to zero, the corresponding temperature is the stability limit. 

### 3.3. Stability Analysis Result

Combined with the traditional elastic design method, the stability limits of the concave and convex models for TBCs were analyzed with different radius curvatures and layer thicknesses based on the cylinder model.

#### 3.3.1. Effect of Curvature on Stability Limit

The effects of the Sub radius curvature on the elastic limit and the stability limit for the concave and convex model are shown in Table 2. The stability limits are greater than the elastic limit for both the concave and convex models. The elastic limit and stability limit increase with increasing radius curvature. The elastic and stability limits for the convex model are higher than those in the concave model under the same radius curvature. With the development of research and manufacturing levels for TBCs, the actual working temperature has reached more than 1000 °C; however, the stability limit obtained in this study is less than the actual temperature, as shown in Table 2 (just between 800 and 900 °C). It is mainly due to stress release and relaxation under high temperatures as an influence of material creep, which releases system stress effectively. However, creep is not considered in this study, which results in the calculated thermal stresses being higher than those in actual conditions and having smaller stability limits.

#### 3.3.2. Effect of Layer Thickness on Stability Limit

The effects of layer thickness on elastic limit and stability limit for the concave and convex model are shown in Table 3 and Table 4, respectively. First, the sensitive relationship between the layer thickness and elastic limit, and stability limit can be obtained, and the effect of thickness of the TC layer on the elastic and stability limits is greater than that of the BC layer thickness variation. Second, the elastic limit and stability limit increase with a thickening layer, and the slope of the stability limit curve decreases gradually, which indicates that, with an increase in TC thickness, the incremental limit load of TBCs decrease. Even with the TC thickness increasing to the threshold, the incremental value is very small. This indicates that trying the limit load cannot be improved by increasing the thickness of TC.

Hence, the elastic and stability limits for the convex model are higher than those in the concave model, as shown in Table 2, Table 3 and Table 4. Namely, compared with the outer convex model, the concave model has less ultimate bearing capacity, and the TBCs are usually damaged at this position, which is in agreement with previous experimental results, as shown in Figure 1. The crack initiation around the concave interface after a long working time leads to spalling failure.

## 4. Constant Temperature Oxidation Test and Thermal Shock Test

To verify the rationality of the stability limit analysis model and the results of multilayer structures, according to the testing methods for thermal cycle and thermal shock resistance of thermal barrier coatings (standard NO: JIS H8451-2008), a series of 8-YSZ TBC specimens were prepared by the plasma spraying process and combined with the constant temperature oxidation test and thermal shock test. The TBC specimens were stable when the thermal shock temperatures were 850 °C and 950 °C, and the structure can be considered to be in a stable state. When the thermal shock temperature was 1050 °C, the stability of the TBC samples was significantly reduced and the stability limits were between 950 °C and 1050 °C.

### 4.1. Specimen Preparation

Classical 8-YSZ TBC specimens were prepared, and the dimensions and materials for the TBCs are shown in Table 5. 

**Figure 2:**
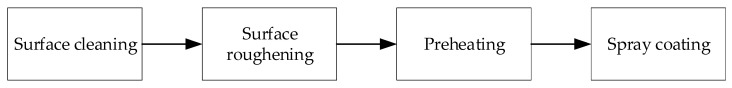
The specimen preparation process.

**Figure 3:**
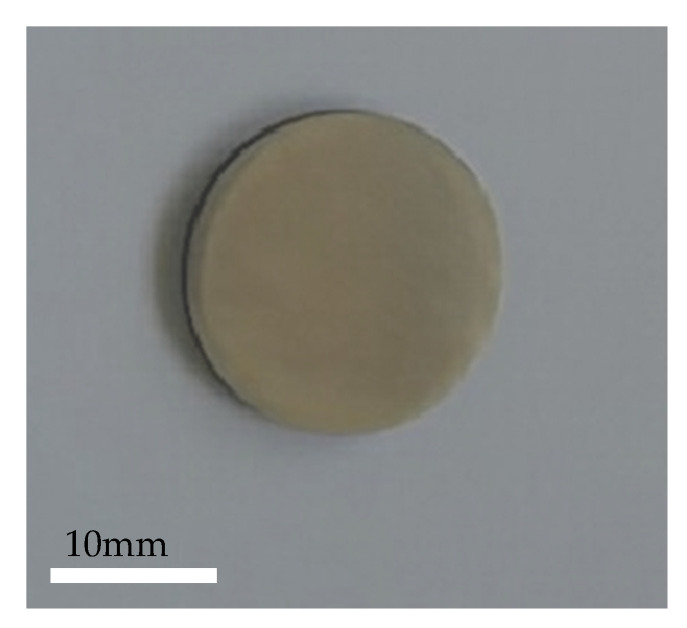
The specimen after deposition.

The specimen preparation process is shown in Figure 2, and the specimen after deposition is shown in Figure 3.

### 4.2. Testing Plan and Processing

For the constant temperature oxidation test, 24 TBC samples were heated to 1000 °C in an SRJX-8-13 box-type resistance furnace, and then, at 1 h, 3 h, 6 h, 12 h, 24 h, 48 h, 72 h, and 96 h, three samples were taken out each time and air-cooled. To study the stability of TBCs under a thermal cycle, thermal shock tests were carried out at different temperatures based on the failure evaluation of coat peeling. The thermal shock temperatures were 850 °C (three samples, numbered as T1-1, T1-2, and T1-3), 950 °C (three samples, numbered as T2-1, T2-2, and T2-3), and 1050 °C (five samples, numbered as T3-1, T3-2, T3-3, T3-4, and T3-5), for a total of 11 samples. The heat preservation time of each cycle was 1 h, followed by air cooling. Based on the number of thermal shocks and the thermal shock temperature used [32,33], the upper limit of the thermal shock number was set as 300 times; after that, if still no cracking or spalling failure occurs, we argue that the coating structure is stable. When the thermal shock temperature is 1050 °C, a sample is taken out at the 5th and 30th hours to investigate the difference in interface deformation and to verify the stability of interface displacement.

### 4.3. Test Results and Verification

#### 4.3.1. Verification of Interface Stability

The TGO layer formed at the TC/BC interface and was distributed in a strip at the beginning of the thermal cycle at 1050 °C. After 30 thermal cycles, despite the TC layer not spalling, micro-cracks appeared in the coating internal. Once the micro-cracks were connected and extended, an interface crack appeared. As the heat cycle continued, the sample started to peel [32], failures were located at the TC/TGO and TGO/BC interfaces, and the cracking direction was parallel to the interface direction. The failure mechanism was determined to be in accordance with the conclusions of similar literature [33], which verified previous conclusions about the cracking location of the coating.

The TBCs prepared by plasma spraying were not flat at the initial SUB/BC/TC interfaces. Under thermal shock conditions, the TGO layer formed and continuously thickened at the BC/TC interface due to BC oxidation. At the same time, the unfitness of the interface was further aggravated (the amplitude of the curve interface changes). The interface of the TBCs showed significant instability (displacement instability) until internal cracks appeared and further expanded the coating, eventually leading to peeling failure of the coating. Since the thermal cycle test is a process with alternating cold and heat, the alternating stress generated in the thermal cycle process makes it easier for the TC layer and TGO layer to generate defects such as holes and micro-cracks. After several thermal cycles, defects such as holes in the coating gradually grow and connect to form small cracks. The micro-cracks further expand and connect under the actions of stress and penetrate through the coating, resulting in peeling of the coating. During thermal shock, some cracks appear at the TC/TGO/BC interface, and the failure can be observed after the peeling layer is removed. This is mainly due to the presence of fluctuation in the interface topography of TC/TGO/BC. Under thermal cycling conditions, BC layer oxidation, thermal mismatch, phase transition, and other factors cause a very complex stress state near the interface. Especially for the convex part of the interface morphology, its thermal stress state is more complex. Under the condition of alternating heat and cold during the thermal cycling process, the stress concentration in the above parts is more likely to be generated, and the residual stresses are usually large, which further promote the generation and extension of the crack defect in the coating until the final peeling failure. Therefore, the interfacial oxidation growth brought by the thermal cycle will cause a deformation in the coating interface to reduce the stability and then will lead to coating peeling failure, which is consistent with the above conclusions about the overall stability of the coating and the interfacial stability.

#### 4.3.2. Verification of the Analysis Method for Structural Stability Limit

The stability limit range of TBCs can be verified by a thermal shock test. As shown in Table 6, due to the relatively low-temperature oxidation at 850 °C, the three samples numbered T1-1, T1-2, and T1-3 show no significant peeling or cracks on the coating surface after 300 thermal shocks. At the thermal shock temperature of 950 °C, no obvious coating spalling or significant cracks appear in samples T2-1 and T2-3 after 300 thermal shocks. However, sample T2-2 endured thermal shocks only 154 times before cracks appear on the coating surface. At the thermal shock temperature of 1050 °C, sample numbered T3-1 showed no obvious coating peeling or significant cracks after 300 thermal shocks, but significant cracks appeared on the coating surface after 77 thermal shocks for sample T3-2 and after 139 thermal shocks for sample T3-3.

From the test results, the effects of thermal shock temperature on the failure of TBCs are more obvious at the high-temperature stage (such as 1050 °C). For the thermal shock test conditions, the TBCs have a thermal stability limit. Additionally, for the thermal shock temperatures 850 °C, 950 °C and 1050 °C, regardless of the coating preparation, test conditions, and precision of the instrument, the temperatures 850 °C and 950 °C can be assumed to be stable temperatures, while the thermal barrier coating exhibited poor stability under thermal cycling at 1050 °C. Therefore, it can be considered that the stability limit of the thermal barrier coating prepared in this study is between 950 °C and 1050 °C, slightly higher than the results obtained from the structural stability limit analysis model in the previous section (800–950 °C). This is mainly due to the simplification of some factors in the stability limit analytical model, which causes specific errors. Although the thermal shock tests are only carried out at three temperature conditions, it verified the validity of the stability limit analysis model presented in this study.

## 5. Conclusions

Considering the characteristics of the microstructure interface morphology of TBCs, an analytical method for multiplayer structure shakedown analysis was established, and the isothermal and thermal shock tests for 8-YSZ TBCs specimen were carried out to verify the validity of the proposed method. The main conclusions included the following:

For classical TBCs, the stability limit is significantly greater than the elastic limit, and the limit for the convex model was higher than that in the concave model. Additionally, the larger the radius of curvature of the Sub and the thickness of the TBCs, the higher the stability limit. This indicates that the first failure occurs in the concave area, and a smooth interface for the coating is beneficial to the stability of the multiplayer structure. According to the experimental results, (i) the interface oxidation at high temperature would cause interface instability, which will further affect the overall stability and lead to coat peeling failure; (ii) he mathematical approach developed for the stability limit evaluation is reliable and reasonable; (iii) the stability of the TBCs was significantly reduced with the increase in temperature; and (iv) for the coating sample prepared this time, when the thermal shock temperatures were 850 °C and 950 °C, the TBC sample had good stability and the structure could be considered to be in a stable state, and when the thermal shock temperature was 1050 °C, the stability of the TBC samples was significantly reduced, and the shakedown limit was between 950 °C and 1050 °C, which verifies the effectiveness of the shakedown limit analysis model of the multi-layer structures proposed in this paper.

## Figures and Tables

**Table 1:** The bilinear plastic date for BC [26,27].

T/°C	400	400	600	600	800	800	900	900	1000	1000
σ/MPa	1100	2500	1100	2200	300	380	45	60	10	15
ε_p_	0	0.24	0	0.30	0	0.02	0	0.02	0	0.01

**Table 2:** Limit load for TBCs with different curvatures of Sub.

Radius of Curvature/mm	Concave Elastic Limit Load/°C	Concave Stability Limit Load/°C	Convex Elastic Limit Load/°C	Convex Stability Limit Load/°C
15.165	605	797.8	722	949.3
17.165	609	799.8	724	950.1
19.165	615	802.8	726	950.9
21.165	623	806.8	728	951.6
23.165	635	812.8	730	952.3

**Table 3:** Relationship between limit load and thickness of TBCs for the concave model.

Thickness Incremental Ratio	TC Thickening Elastic Limit Load/°C	BC Thickening Elastic Limit Load/°C	TGO Thickening Elastic Limit load/°C	TC Thickening Stability Limit Load/°C	BC Thickening Stability Limit Load/°C	TGO Thickening Stability Limit Load/°C
0	605	605	605	797.8	797.8	797.8
0.1	622	605.1	605.4	805.3	798	798.14
0.2	636	605.2	606	813.6	798.2	798.4
0.3	648	605.3	606.5	822.7	798.35	798.7

**Table 4:** Relationship between limit load and thickness of TBCs for the convex model.

Thickness Incremental Ratio	TC Thickening Elastic Limit Load/°C	BC Thickening Elastic Limit Load/°C	TGO Thickening Elastic Limit load/°C	TC Thickening Stability Limit Load/°C	BC Thickening Stability Limit Load/°C	TGO Thickening Stability Limit Load/°C
0	730	730	730	949.3	949.3	949.3
0.1	743	731.2	731.9	952.8	949.5	950.1
0.2	754	732.4	733.5	956.4	949.7	950.9
0.3	763	733.6	735.5	960	949.9	951.4

**Table 5:** The dimensions and materials for the specimens.

	SUB	BC	TC
Material	DZ 125	Ni-Cr-Al-Yttrium	8 wt%Y_2_O_3_-ZrO_2_
Radius/mm	10	10	10
Thickness/mm	2	0.1	0.25

**Table 6:** Failure thermal cycles of coatings at different thermal shock temperatures.

Sample	850 °C			950 °C			1050 °C		
	T1-1	T1-2	T1-3	T2-1	T2-2	T2-3	T3-1	T3-2	T3-3
Thermal cycles	300 *^)^	300 *^)^	300 *^)^	300 *^)^	154	300 *^)^	300 *^)^	77	139

Note: *^)^ No peeling or cracking was observed after 300 thermal cycles.

## Data Availability

Not applicable.

## References

[1] Ziming Y., Brian G., James G., Deivanayagam H., John S., Carl S., Felipe C., Sanjay S., Benjamin L. (2021). A comprehensive experimental investigation of low-temperature combustion with thick thermal barrier coatings. Energy.

[2] Sait F., Gurses E., Aslan O. (2020). Modeling and simulation of coupled phase transformation and stress evolution in thermal barrier coatings. Int. J. Plast..

[3] Liu Y.Z., Zheng S.J., Zhu Y.L., Wei H., Ma X.L. (2016). Microstructural evolution at interfaces of thermal barrier coatings during isothermal oxidation. J. Eur. Ceram. Soc..

[4] Padture N.P., Gell M., Jordan E.H. (2002). Thermal Barrier Coatings for Gas-Turbine Engine Applications. Science.

[5] He M.Y., Hutchinson J.W., Evans A.G. (2003). Simulation of stresses and delamination in a plasma-sprayed thermal barrier system upon thermal cycling. Mater. Sci. Eng. A.

[6] Karlsson A.M., Hutchinson J.W., Evans A.G. (2002). A fundamental model of cyclic instabilities in thermal barrier systems. J. Mech. Phys. Solids.

[7] Mondal K., Nuñez L., Downey C.M., van Rooyen I.J. (2021). Thermal Barrier Coatings Overview: Design, Manufacturing, and Applications in High-Temperature Industries. Ind. Eng. Chem. Res..

[8] Kyaw S., Jones A., Hyde T. (2013). Predicting failure within TBC system: Finite element simulation of stress within TBC system as affected by sintering of APS TBC, geometry of substrate and creep of TGO. Eng. Fail. Anal..

[9] Zheng X.T., Cheng S., Feng L., Yu J.-Y., Chen Y. (2013). Shakedown analysis of the pressure piping bend under cycle moving thermal loadings. Press. Vessel. Technol..

[10] Zheng X.T., Peng C.F., Yu J.Y., Wang C. (2013). Shakedown analysis of U-groove butt welded joints under cyclic thermo-mechanical loadings. Trans. China Weld. Inst..

[11] Gokhfeld D.A., Charniavsky O.F. (1982). Limit Analysis of Structures at Thermal Cycling. J. Appl. Mech..

[12] Xue M.D., Wang X.F., Williams F.W., Xu B.Y. (1997). Lower-bound shakedown analysis of axisymmetric structures subjected to variable mechanical and thermal loads. Int. J. Mech. Sci..

[13] Chen H. (2010). Lower and Upper Bound Shakedown Analysis of Structures With Temperature-Dependent Yield Stress. J. Press. Vessel Technol..

[14] Liu X., Feng X. (1991). Shakedown analysis of a thick-walled cylindrical tube considering the material property changing with temperature. Eng. Mech..

[15] Du S.T., Xu B.Y. (1990). A Kinematical Shakedown Theorem Considering External Loading and Temperature Variation. Acta Mech. Solida Sin..

[16] Du S.T., Liu H.B., Chen S.H., Lian J.S. (1995). Shakedown Analysis of Elasto—Plastic Structures Subjected to External Loading and Temperature Variation. Appl. Math. Mech..

[17] Evans H.E. (2011). Oxidation failure of TBC systems: An assessment of mechanisms. Surf. Coat. Technol..

[18] Mumm D.R., Evans A.G., Spitsberg I.T. (2001). Characterization of a cyclic displacement instability for a thermally grown oxide in a thermal barrier system. Acta Mater..

[19] Monteverde F., Savino R., Fumo M., Maso A.D. (2010). Plasma wind tunnel testing of ultra-high temperature ZrB_2_–SiC composites under hypersonic re-entry conditions. J. Eur. Ceram. Soc..

[20] Karlsson A.M., Evans A.G. (2001). A numerical model for the cyclic instability of thermally grown oxides in thermal barrier systems. Acta Mater..

[21] Hille T.S., Turteltaub S., Suiker A.S.J. (2011). Oxide growth and damage evolution in thermal barrier coatings. Eng. Fract. Mech..

[22] He M.Y., Evans A.G., Hutchinson J.W. (2000). The ratcheting of compressed thermally grown thin films on ductile substrates. Acta Mater..

[23] Monti R., Fumo M.D.S., Savino R. (2006). Thermal Shielding of Reentry Vehicle by Ultra-High-Temperature Ceramic Materials. J. Thermophys. Heat Transf..

[24] Xu Y.-Q., Li S.-J., Yang X.H., Li J.-F. (2009). Numerical simulation of structural instability in thermal barrier system during thermal cycling. Mach. Des. Manuf..

[25] Huang X., Ding J., Zhou J., Zeng X. (2011). Numerical Simulation of Interfacial Displacement Instability in TBC System. Surf. Technol..

[26] Aktaa J., Sfar K., Munz D. (2005). Assessment of TBC systems failure mechanisms using a fracture mechanics approach. Acta Mater..

[27] Rösler J., Bäker M., Aufzug K. (2004). A parametric study of the stress state of thermal barrier coatings: Part I: Creep relaxation. Acta Mater..

[28] Mao W.G., Zhou Y.C., Yang L., Yu X.H. (2006). Modeling of residual stresses variation with thermal cycling in thermal barrier coatings. Mech. Mater..

[29] Zhou C., Na W., Xu H. (2007). Comparison of thermal cycling behavior of plasma-sprayed nanostructured and traditional thermal barrier coatings. Mater. Sci. Eng. A.

[30] Li M.Y., Zhang C.Y., Sun X.F., Gong S.K., Hu W.Y., Guan H.R., Hu Z.Q. (2002). Failure Mechanism of EB-PVD Thermal Barrier Coating Subjected to Thermal Cycling. J. Mater. Eng..

[31] Li M.H., Sun X.F., Zhang Z.Y., Gong S.K., Hu Z.Q. (2002). Oxidation and phase structure of the bond coat in EB-PVD thermal barrier coatings during thermal cycling. Acta Metall. Sin. Chin. Ed..

[32] Chen L.Y., Li Y., Jiang H. (2007). General computation method for axisymmetric temperature and thermal stress of multilayer cylinder. J. Mater. Metall..

[33] Tolpygo V.K., Clarke D.R. (2000). Surface rumpling of a (Ni, Pt)Al bond coat induced by cyclic oxidation. Acta Mater..

